# Resources for the practice of pediatric neuro-oncology in Mexico: a cross-sectional evaluation

**DOI:** 10.3389/fonc.2024.1330705

**Published:** 2024-06-21

**Authors:** Daniela Arce-Cabrera, Gabriela Escamilla-Asiain, Melisa F. Nájera-Castillo, Regina M. Navarro-Martín del Campo, Mariana Ortiz-Azpilcueta, Francisco J. Pantoja-Guillén, Farina E. Arreguín González, Imelda Zapata-Sosa, Jocelyn Z. Lugo-Juárez, Daniel Santillán Cortéz, Andrés Morales-La Madrid, Daniel C. Moreira, Alma E. Benito-Reséndiz

**Affiliations:** ^1^ Pediatric Oncology Department, Sinaloa Pediatric Hospital, Culiacan, Sinaloa, Mexico; ^2^ Pediatric Oncology Department, Teletón Children’s Hospital of Oncology, Querétaro, Querétaro, Mexico; ^3^ Pediatric Oncology Department Hospital for Children of Toluca. Maternal and Child Institute of the State of Mexico, Toluca, Estado de Mexico, Mexico; ^4^ Pediatric Oncology Department, Civil Hospital of Guadalajara Dr. Juan I. Menchaca, Guadalajara, Jalisco, Mexico; ^5^ Pediatric Oncology Department, Pediatric Hospital of the National Medical Center 21st century Mexican Social Security Institute (IMSS), Mexico City, Mexico; ^6^ Pediatric Oncology Department, Agustín O’Horán General Hospital, Merida, Yucatan, Mexico; ^7^ Pediatric Oncology Department, National Medical Center November 20 ISSSTE, Mexico City, Mexico; ^8^ Pediatric Oncology Department, Zacatecas General Hospital, Zacatecas, Zacatecas, Mexico; ^9^ Barcelona Pediatric Oncology Center, Saint Joan de Déu Hospital, Barcelona, Spain; ^10^ Department of Global Pediatric Medicine, St. Jude Children’s Research Hospital, Memphis, TN, United States

**Keywords:** LMIC, pediatric neuro-oncology, pediatric brain tumors, care capacity, resource availability

## Abstract

**Background:**

The evaluation of existing resources and services is key to identify gaps and prioritize interventions to expand care capacity for children with central nervous system (CNS) tumors. We sought to evaluate the resources for pediatric neuro-oncology (PNO) in Mexico.

**Methods:**

A cross-sectional online survey with 35 questions was designed to assess PNO resources and services, covering aspects including number of patients, infrastructure, human resources, and diagnostic and treatment time intervals. The survey was distributed to the members of the Mexican Association of Pediatric Oncology and Hematology (AMOHP) who belong to the nation’s many different health systems.

**Results:**

Responses were obtained from 33 institutions, distributed throughout the country and part of the many health systems that exist in Mexico. Twenty-one (64%) institutions had less than 10 new cases of pediatric CNS tumors per year. Although 30 (91%) institutions saw pediatric patients up to the age of 18 years, 2 (6%) had a cutoff of 15 years. Twenty-four (73%) institutions had between 1 and 3 pediatric oncologists providing care for children with CNS tumors. Six (18%) institutions did not have a neurosurgeon, while 19 (57%) institutions had a pediatric neurosurgeon. All centers had a pathology department, but 13 (39%) institutions only had access to basic histopathology. Eleven (33%) institutions reported histopathological diagnoses within one week, but 3 (9%) took more than 4 weeks. Radiotherapy for pediatric CNS tumors was referred to outside centers at 18 (55%) institutions. All centers had access to conventional cytotoxic chemotherapy, but only 6 (18%) had access to targeted therapy. Eighteen (55%) respondents estimated a survival rate of less than 60%. Fifteen (45%) centers attributed the main cause of mortality to non-tumor related factors, including infection and post-surgical complications.

**Conclusions:**

This is the first national assessment of the resources available in Mexico for the treatment of CNS tumors. It shows disparities in resource capacity and a lack of the specific and efficient diagnoses that allow timely initiation of treatment. These data will enable the prioritization of collaborative interventions in the future.

## Introduction

Central nervous system (CNS) tumors are the second most common pediatric cancer globally (1). Importantly, CNS tumors in children and adolescents have high mortality and morbidity rates (2). In low- and middle-income countries, late presentation and limited infrastructure for comprehensive care lead to significantly lower survival rates. Although historically the study of pediatric CNS tumors has not been prioritized, recent attention has been brought to the disparities in care available and outcomes (3–5).

In Mexico, each year, approximately 850 cases and 300 deaths occur for CNS tumors in children and adolescents less than 19 years-of-age (6). Mexico is an upper-middle-income country in North America with 37 million inhabitants under the age of 18 years (7). In the country, numerous public and private healthcare systems exist in parallel (8). The public sector is primarily funded through the government, providing services at no direct cost to the patient, and includes organizations such as the Mexican Social Security Institute (IMSS) and the Institute for Social Security and Services for State Workers (ISSSTE). Only approximately 5% of Mexicans have private health insurance. Since the early 2000s, Mexico has sought to have universal health coverage through government funded initiatives (9). In 2020, Mexico implemented the Institute of Health for Well-being (INSABI) to expand free healthcare coverage, replacing the Seguro Popular that was established in 2003.

The nation’s federal programs have recognized childhood cancer as an important part of child health and offered access to treatment for children with cancer by covering the cost of therapy. Despite these efforts, the systems often struggle with underfunding and inequality in service quality (10). Specifically, the 5-year net survival of pediatric CNS tumors in Mexico is estimated to be approximately 37% (2). Recent publications suggest that poor outcomes are associated with high rates of surgical morbidity, treatment-related mortality, and abandonment (11, 12).

There are approximately 70 pediatric cancer units in Mexico, with varying infrastructure and resources (13). The care of children with CNS tumors requires access to complex infrastructure and the availability of numerous pediatric subspecialists (14). The evaluation of existing resources and services is key to identify gaps and prioritize interventions to expand care capacity for this vulnerable patient population. Efforts to describe the resources for pediatric neuro-oncology have been made in countries in Latin America, but these have not included Mexico. An analysis from Chile demonstrated access to basic services to provide care for children with CNS tumors, while one from Paraguay described more limited available infrastructure (15–17). In this study, we sought to evaluate the resources for the practice of pediatric neuro-oncology (PNO) in Mexico.

## Methods

### Study design and participants

A cross-sectional online survey with 35 questions was designed to assess pediatric neuro-oncology resources and services (Appendix 1). Survey questions were initially created by the first author and subsequently revised by the research team. The questions covered aspects including number of patients, infrastructure, human resources, and diagnostic and treatment time intervals. Survey questions were created as multiple-choice and open-text field questions. The survey was distributed to the members of the Mexican Association of Pediatric Oncology and Hematology (AMOHP) and was open from February 1^st^ to 16^th^, 2023. Participation in the survey was voluntary and no personal identifying information was collected.

### Statistical analysis

Descriptive statistics were used to analyze all results. For these analyses, SPSS^®^ version 22 was used.

## Results

### Responding institutions

**Figure 1:**
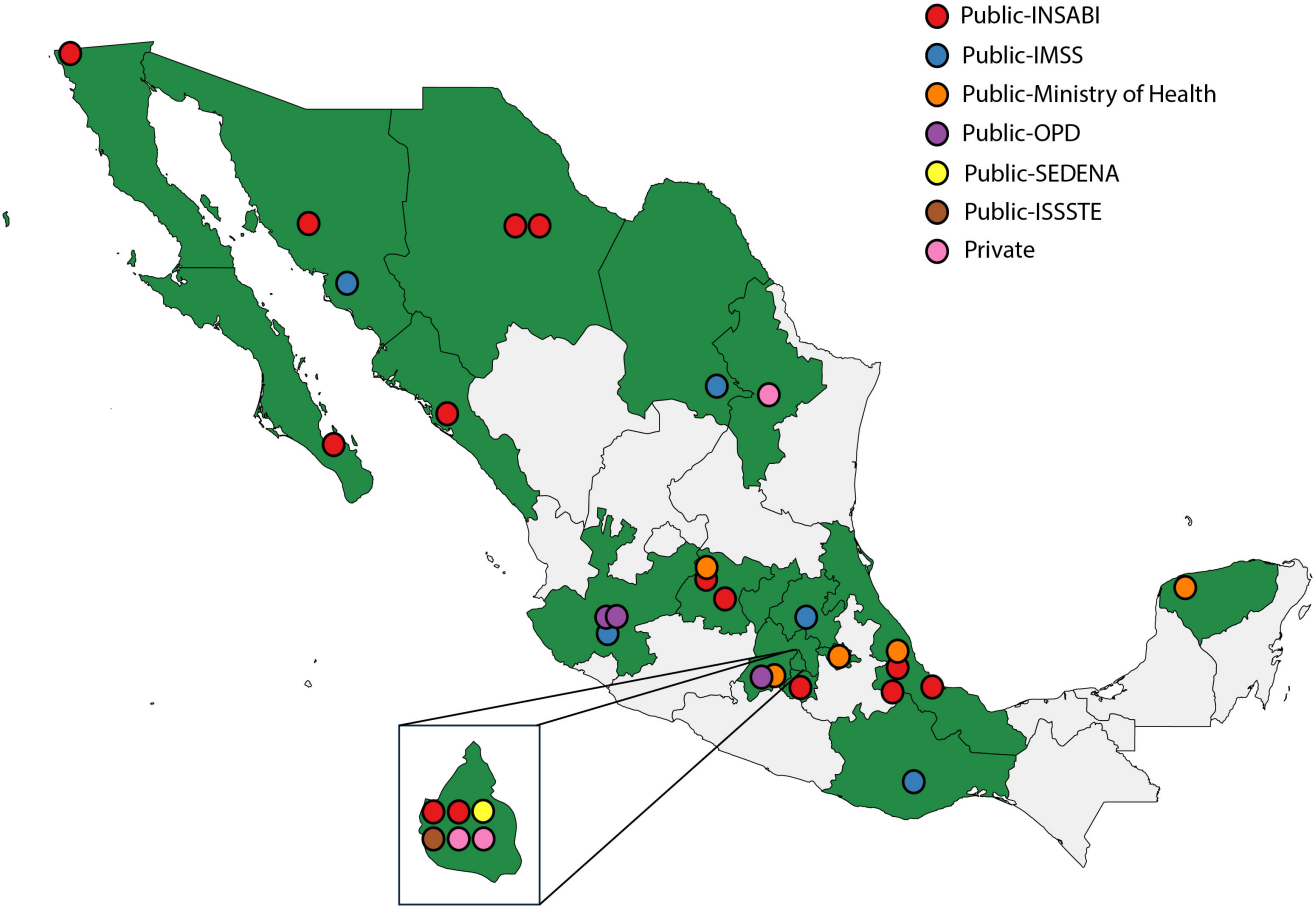
Geographic distribution of responding institutions and health systems.

Overall, responses were obtained from 33 institutions distributed throughout Mexico (**Figure 1**). Institutions are part of the many health systems that exist in Mexico (**Table 1**). Twenty-one (64%) institutions had less than 10 new cases of pediatric CNS tumors per year. In addition, although 31 institutions (94%) saw pediatric patients up to the age of 18 years, 2 (6%) had a cutoff of 15 years. In 14 (42%) centers, the initiation of the diagnostic approach for children with CNS tumors was carried out by a pediatric oncologist. In 12 (36%) centers, it was carried out by neurology or neurosurgery teams, while in 6 (18%) centers it was carried out by general pediatrics.

**Table 1:** Hospital characteristics.

Characteristic	n (%)
Region	
*North*	3 (9)
*Northeast*	1 (3)
*Pacific coast*	3 (9)
*Bajío*	5 (15)
*West*	2 (6)
*Central*	11 (33)
*Gulf*	7 (21)
*South*	1 (3)
Healthcare system	
*Public-IMSS*	5 (15)
*Public-ISSTE*	1 (3)
*Public-Ministry of Health*	5 (15)
*Public-INSABI*	14 (42)
*Public-SEDENA*	1 (3)
*Public-OPD*	3 (9)
*Private*	4 (12)
New pediatric CNS tumors per year	
*<10*	21 (64)
*10–20*	7 (22)
*21–30*	2 (6)
*>30*	3 (9)
Maximum age of pediatric services	
15 years	2 (6)
18 years	30 (91)
21 years	1 (3)

### Infrastructure and resources

Twenty-four (73%) institutions had between 1 and 3 pediatric oncologists providing care for children with CNS tumors (**Table 2**). Although 2 (6%) institutions did not have a neurosurgeon, 19 (57%) institutions had a pediatric neurosurgeon. Twenty-three (70%) centers performed second-look surgeries to achieve larger tumor resections.

**Table 2:** Hospital resources and infrastructure.

Characteristic	n (%)
Pediatric oncologists per hospital	
*1–3*	24 (73)
*4–5*	5 (15)
*>5*	4 (12)
Neurosurgeons per hospital	
*Only adult neurosurgeon*	8 (24)
*Pediatric neurosurgeon*	15 (45)
*Adult and pediatric neurosurgeon*	4 (12)
*None*	6 (18)
Radiation oncologists per hospital	
*Adult radiation oncologists*	18 (55)
*Pediatric radiation oncologists*	15 (45)
ICU bed availability	
*None*	4 (12)
*1–3*	2 (6)
*4–6*	10 (30)
*7–9*	7 (21)
*>10*	10 (30)
Imaging availability	
*CT at facility*	29 (88)
*CT at outside facility*	1 (3)
*MRI*	18 (55)
*MRI at outside facility*	13 (39)
Pathology	
*Basic histopathology*	13 (39)
*Basic histopathology and immunohistochemistry*	13 (39)
*Basic histopathology, immunohistochemistry, and basic molecular testing*	7 (21)
Radiotherapy	
*At the center*	15 (45)
*Referred to another center*	18 (55)

Four (12%) institutions had no pediatric intensive care unit (PICU). Where such a unit was available, the number of beds was scarce and only 16 (48%) centers had 24-hour specialist coverage in the PICU. Twenty-one (63%) centers reported that children who undergo surgery for CNS tumors have priority access to the PICU.

All centers had a pathology department, but 13 (39%) had only basic histopathological testing. At 15 (45%) institutions, radiation oncologists with expertise in pediatric radiotherapy were available. Furthermore, radiotherapy for pediatric CNS tumors was referred to outside institutions in 18 (55%) institutions.

All centers had access to conventional cytotoxic chemotherapy, but only 6 (18%) had access to targeted therapy. Furthermore, 17 (58%) centers relied on non-governmental organizations (NGOs) and foundations to offset the cost of cancer-directed medications. Although 29 (88%) centers had a blood bank and access to blood products, there were 4 (12%) centers without these services. Nineteen (58%) centers had a pediatric palliative care service, while the remaining 14 (42%) did not, with pediatric oncologists providing these services or through other solutions.

### Timelines for diagnosis and treatment

**Figure 2:**
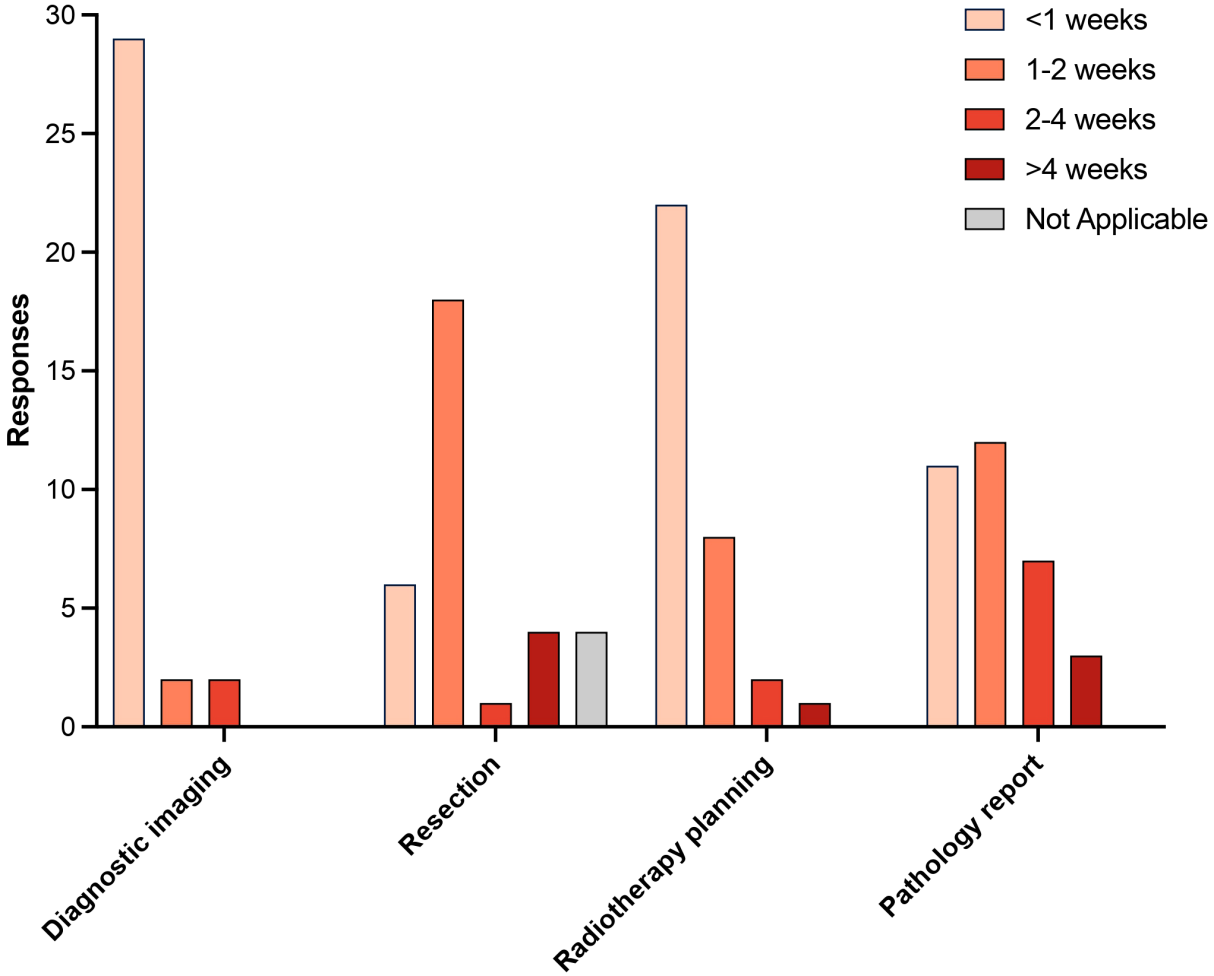
Diagnostic and treatment intervals.

The diagnostic and treatment intervals are included in **Figure 2**. At 29 (88%) institutions, imaging for the diagnosis and follow up of CNS tumors could be obtained within 1 week. When a resection was needed, this could happen in less than a week at 6 (18%) centers. Furthermore, radiotherapy planning could occur in less than a week at 22 (67%) institutions. Although 11 (33%) centers had a histopathological diagnosis in one week, 3 (9%) did not have pathology reports available until more than 4 weeks.

### Outcomes

Understanding that most centers did not have comprehensive hospital-based cancer registries, respondents were asked to estimate 5-year overall survival for children with CNS tumors. Eighteen (55%) respondents estimated a survival of less than 60% (**Table 3**). Furthermore, when asked about the causes of mortality, 15 (45%) centers attributed the main cause of mortality to non-tumor related factors, such as infection and post-surgical complications.

**Table 3:** Perceived outcomes.

Characteristic	n (%)
Estimated 5-year survival	
*≤50%*	6 (18)
*51–60*	12 (36)
*61–70*	7 (21)
*>70*	2 (6)
*Unknown*	6 (18)
Main cause of mortality	
*Tumor*	16 (48)
*Infections*	9 (27)
*Post-surgical*	6 (18)
*Unknown*	2 (6)

## Discussion

This study sought to evaluate the elements that lead to comprehensive pediatric neuro-oncology care in Mexico. Our data suggest significant limitations and disparities in resources, prolonged timelines in key elements of care, and poor outcomes.

Ideally, hospitals that treat children with CNS tumors should have all the necessary resources to carry out comprehensive diagnosis and treatment, including not only cancer-directed therapy, but also supportive and palliative care. Furthermore, these resources need to be integrated into functioning, efficient pediatric neuro-oncology services (18). Our data suggest that, for many institutions, key elements of care are lacking. Moreover, the evaluation of time intervals of care, with delayed times for some of the core elements of diagnosis and treatment, suggests that optimization of service integration is a priority.

The fragmented healthcare system in Mexico translates into unequal resources and different packages of coverage for patients. This is reflected in our data based on the variability in described resources. For example, in 12% of the centers there was no PICU available for patients, as well as limited specialists for the postoperative care of children with CNS tumors. This care context increases the risk of postoperative complications, one of the most common causes of mortality described by respondents. Investments in post-operative care and infection control may be one of the priority interventions to improve outcomes for many centers in Mexico. These represent immense areas of opportunity to reduce mortality, in many cases with limited investment (19).

In the era of a rapidly evolving field of pediatric neuro-oncology based on molecular characterization and risk-stratification, strategies to expand diagnostic capacity are essential (20). Many of the included centers have only basic pathology and incur in important delays in reporting. The regionalization of pathologic evaluation for pediatric CNS tumors would be a strategy to optimize the available resources (21). With a more comprehensive diagnostic infrastructure, novel approaches and treatment would become more relevant. Importantly, only a small number of centers had access to targeted therapies, so it is also necessary to implement strategies to expand access to novel therapeutics for all centers.

The included centers had limited capacity to estimate survival for children with CNS tumors. The World Health Organization has encouraged the development of cancer registries as a step toward pediatric cancer control (22). Cancer registries provide invaluable information about disease burden and help establish priorities for cancer in low-resource settings. Expanding hospital-based cancer registries would establish a framework for more data on clinical characteristics and outcomes, helping define evidence-based strategies to improve services.

This study has multiple limitations. Firstly, data was collected from less than half of the institutions caring for children with cancer in Mexico. Although we captured data on institutions in different geographic areas and health systems, there may be additional insight that was not elucidated. Secondly, although we sought to evaluate the multiple elements that are needed to provide care for children with CNS tumors, a more in-depth evaluation would be needed to define detailed strategies to expand access to quality care for children treated at these institutions. In addition, the existence of tumor boards and collaborations focused on PNO were not collected in the survey. Finally, although elements of perceived survival and outcomes were collected, patient-level data was not collected. Retrospective or prospective data collection would be needed to provide more reliable survival estimates.

This study represents the first description of the resources for PNO care in Mexico, generating a vision of the essential needs to provide comprehensive, quality care for children with CNS tumors. The work has galvanized the integration of a group of Mexican pediatric oncologists especially invested in strategies to expand quality care for children with CNS tumors. Progress must be made in the development of innovative methods of diagnosis, treatment, and long-term follow-up with the aim of improving survival rates and reducing treatment-related toxicity.

## Data availability statement

The original contributions presented in the study are included in the article/**Supplementary Material**. Further inquiries can be directed to the corresponding author.

## Author contributions

DA: Data curation, Software, Writing – original draft. GE: Conceptualization, Data curation, Investigation, Writing – original draft. MN: Investigation, Writing – original draft. RN: Conceptualization, Investigation, Writing – original draft. MO: Conceptualization, Investigation, Writing – original draft. FP: Conceptualization, Writing – original draft. FA: Conceptualization, Data curation, Investigation, Writing – original draft. IZ: Conceptualization, Investigation, Writing – original draft. JL: Conceptualization, Investigation, Writing – original draft. DS: Data curation, Formal analysis, Methodology, Visualization, Writing – original draft. AM: Conceptualization, Formal analysis, Validation, Writing – original draft. DM: Supervision, Validation, Writing – review & editing. AB: Formal analysis, Methodology, Supervision, Visualization, Writing – original draft, Project administration, Validation, Writing – review & editing.
