# Supplementary material for: Resources for the practice of pediatric neuro-oncology in Mexico: a cross-sectional evaluation
Source: Front Oncol. 2024 Jun 21;14:1330705. doi: 10.3389/fonc.2024.1330705 (PMC11224457; doi:10.3389/fonc.2024.1330705)
Supplement: Supplementary file 1 [file DataSheet_1.docx]

**SURVEY QUESTIONS**

1. Name of the hospital
2. Which healthcare system is the hospital part of?
3. Up to what age are cancer patients seen at your institution?
4. How many new cases of CNS tumors do you have at your center each?
5. What are the five most common CNS tumors?
6. Who initiates the diagnostic approach for CNS tumors at your center?
7. On average, how long (in weeks) does it take for patients to arrive at the oncology service once it is known that they have a CNS tumors?
8. How many pediatric oncologists see patients with CNS tumors at your institution?
9. What imaging studies do you have?
10. On average, how long (days) does it take to schedule the first neuroimaging study?
11. Who operates on patients with CNS tumors?
12. What type of surgical interventions/techniques are performed at your hospital?
13. After surgery, in patients who warrant it, at what time do you perform the lumbar puncture?
14. On average, how long does the scheduling of a resection surgery take?
15. Are second look surgeries performed?
16. Where is post-surgical care carried out?
17. Do you have a blood bank?
18. Check the oncology medications you have:
19. If you do not have these medications, how do you acquire them?
20. Do they have radiotherapy?
21. If it is outsourced, where are patients referred and how is radiotherapy financed?
22. Are patients evaluated by pediatric radiation oncologists?
23. Once the request for evaluation has been made, how long on average (days) does it take to receive the first radiotherapy consultation?
24. If you have a Pediatric Intensive Care Unit (PICU), how many beds do you have available?
25. Do you have pediatric intensivists on all shifts?
26. Are patients with CNS tumors given priority in the PICU?
27. Do you have a pathology service?
28. What pathology testing are available?
29. If you do not have a pathology service, where are you send your tissue for pathologic evaluation?
30. How long does it take to receive a histopathological result?
31. Is there a pediatric palliative care service in your center?
32. What specialties do you have for the comprehensive management of patients?
33. If performed, what pre- and post-treatment or pre- and post-surgical evaluations are performed?
34. What is the estimated overall survival of patients with CNS tumors at your center?
35. What are the three most common causes of mortality in patients with CNS tumors?
